# MicroRNAs in Extracellular Vesicles in Sweat Change in Response to Endurance Exercise

**DOI:** 10.3389/fphys.2020.00676

**Published:** 2020-07-15

**Authors:** Sira Karvinen, Tero Sievänen, Jari E. Karppinen, Pekka Hautasaari, Geneviève Bart, Anatoliy Samoylenko, Seppo J. Vainio, Juha P. Ahtiainen, Eija K. Laakkonen, Urho M. Kujala

**Affiliations:** ^1^Gerontology Research Center and Faculty of Sport and Health Sciences, University of Jyväskylä, Jyväskylä, Finland; ^2^Faculty of Sport and Health Sciences, University of Jyväskylä, Jyväskylä, Finland; ^3^Laboratory of Developmental Biology, Faculty of Biochemistry and Molecular Medicine, Biocenter Oulu, University of Oulu, Oulu, Finland; ^4^InfoTech Oulu, Borealis Biobank of Northern Finland, University Hospital, Oulu Center for Cell Matrix Research, Oulu, Finland

**Keywords:** circulating microRNA, sauna, serum, acute exercise response, leukocyte

## Abstract

**Background:**

To date, microRNAs (miRs) carried in extracellular vesicles (EVs) in response to exercise have been studied in blood but not in non-invasively collectable body fluids. In the present study, we examined whether six exercise–responsive miRs, miRs-21, -26, -126, -146, -221, and -222, respond to acute endurance exercise stimuli of different intensities in sweat.

**Methods:**

We investigated the response of miRs isolated from sweat and serum EVs to three endurance exercise protocols: (1) maximal aerobic capacity (VO_2__*max*_), (2) anaerobic threshold (AnaT), and (3) aerobic threshold (AerT) tests. Sauna bathing was used as a control test to induce sweating through increased body temperature in the absence of exercise. All protocols were performed by the same subjects (*n* = 8, three males and five females). The occurrence of different miR carriers in sweat and serum was investigated via EV markers (CD9, CD63, and TSG101), an miR-carrier protein (AGO2), and an HDL-particle marker (APOA1) with Western blot. Correlations between miRs in sweat and serum (post-sample) were examined.

**Results:**

Of the studied miR carrier markers, sweat EV fractions expressed CD63 and, very weakly, APOA1, while the serum EV fraction expressed all the studied markers. In sweat EVs, miR-21 level increased after AerT and miR-26 after all the endurance exercise tests compared with the Sauna (*p* < 0.050). miR-146 after AnaT correlated to sweat and serum EV samples (*r* = 0.881, *p* = 0.004).

**Conclusion:**

Our preliminary study is the first to show that, in addition to serum, sweat EVs carry miRs. Interestingly, we observed that miRs-21 and -26 in sweat EVs respond to endurance exercise of different intensities. Our data further confirmed that miR responses to endurance exercise in sweat and serum were triggered by exercise and not by increased body temperature. Our results highlight that sweat possesses a unique miR carrier content that should be taken into account when planning analyses from sweat as a substitute for serum.

## Introduction

Sweating (perspiration) is the production of water-rich fluid secreted by the skin sweat glands. In humans, sweating is an important part of thermoregulation since the evaporation of sweat from the skin surface lowers body temperature. Sweating thus prevents overheating in hot environments as well as during strenuous exercise. Although sweat has been studied for several decades, it has not established a role as a biomarker source in the field of exercise due to the relatively low abundance of analytes (Mena-Bravo and Luque de Castro, 2014). Thus far, the majority of exercise-associated sweat research has concentrated on ions (Morgan et al., 2004), lactate (Alvear-Ordenes et al., 2005), proteomics, and metabolomics (Harshman et al., 2018), with few RNA-targeted analyses.

MicroRNAs (miRs) are small non-coding RNA-molecules that regulate several biological processes, and they have been identified as essential mediators in exercise adaptations (Sapp et al., 2017). Furthermore, miRs provide a fast response to a changing environment, making them attractive biomarkers to study acute exercise responses (Bartel, 2009; Friedman et al., 2009). There are several circulating miRs (c-miRs) that respond to an acute bout of exercise [for a review, see Silva et al. (2017)]. Of these, miRs-21, -26, -126, -146, -221, and -222 have been shown to respond to an acute endurance exercise stimulus (for a review, see Polakovicova et al., 2016), making them promising biomarkers for exercise monitoring. The available literature suggests that acute bout of aerobic exercise up-regulates miR-21, -126, -146, -221, and -222 and downregulates miR-26 in serum/plasma (Polakovicova et al., 2016). All six c-miRs have been shown to share a role in cellular signaling that underlies exercise adaptations; miRs-21 and -146 are associated with hypoxic adaptations and inflammation (Taganov et al., 2006; Urbich et al., 2008; Krichevsky and Gabriely, 2009), while miRs-26, -126, -221, and -222 are mediators of angiogenesis (Kuehbacher et al., 2007; Fish et al., 2008; Icli et al., 2013).

To facilitate communication between cells and tissues, c-miRs are selectively packed in extracellular vesicles (EVs; Bartel and Chen, 2004; Weber et al., 2010). Extracellular vesicles are small membrane-bound vesicles (from 100 nm to1 μm in diameter) that have the ability to carry information to other cells thereby influencing the recipient cell’s function (Willms et al., 2018). Exosomes, the most abundant subclass of EVs in blood, have a size ranging from 50 to 150 nm. All EVs are composed of a lipid bilayer with embedded transmembrane proteins and a core containing transported signal molecules. C-miR packaging into EVs has been shown to be an active, non-random process (Ratajczak et al., 2006). In addition to EVs, c-miRs in blood can circulate associated with argonaute proteins (AGO) and high-density lipoproteins (HDL; Vickers et al., 2011). Extracellular vesicles in particular are known to act as key mediators in intracellular communication (Denzer et al., 2000).

Intriguingly, EVs and their biomolecule content have been found to be released at the surface of the skin and are therefore, detectable in sweat in addition to blood and other body fluids (Wu and Liu, 2018). The study of Wu and Liu (2018) was the first and thus far only study to examine sweat EVs from healthy humans after aerobic exercise (Wu and Liu, 2018). The authors performed proteomic profiling of sweat exosomes and found sweat proteins to be enriched in EVs. However, there are no studies investigating whether sweat EVs carry miRs.

To date, c-miR’s response to exercise has merely been studied in plasma and serum. Hence, knowledge is lacking concerning the c-miR response to endurance exercise in other body fluids and whether this reflects the response observed in blood. The exercise protocols used in c-miR signaling studies vary greatly (for reviews, see Polakovicova et al., 2016; Sapp et al., 2017), making it difficult to draw solid conclusions on c-miR responses. Furthermore, none of the studies have controlled whether increased body temperature during exercise has a distinct effect on c-miR response.

In this preliminary study, we investigated the occurrence of six exercise-responsive miRs (miR-21, -26, -126, -146, -221, and -222) in sweat and serum EV and non-EV fractions in response to three different endurance exercise tests. Sauna bathing was used as a control for possible responses solely due to increased body temperature. The current study had three main objectives. First, it examined the occurrence of different miR carriers in sweat and serum EV samples. Second, it investigated whether sweat EVs carry miRs and if the miRs are enriched in the EV fraction of sweat. Third, it examined whether the miRs carried in sweat EVs respond to different exercise intensities.

## Materials and Methods

### Study Subjects

Subjects were recruited through University of Jyväskylä email lists and from local sports clubs. Eight healthy subjects (three men and five women) regularly participating in endurance training underwent eligibility screening (structured personal interview of health status, resting electrocardiogram, measurement of blood pressure and assessment of basic blood parameters from venous blood samples) and volunteered to participate in the study. Three subjects reported cross-country skiing as their primary sport while others reported triathlon, rowing, running, and mountain bike orienteering. One subject reported currently participating in various recreational sports. Five subjects reported competing at national and one subject at regional level. For information on training volumes, all subjects completed a training diary beginning from the familiarization period until the end of the last measurement (Table 1, data available for six subjects). All subjects signed written informed consent and the study was approved by the local ethics committee (University of Jyväskylä, Finland).

**TABLE 1:** Subject characteristics (three men and five women) at baseline and training volume based on individual training diaries.

	All (*n* = 8)	Women (*n* = 5)	Men (*n* = 3)
	Mean (SD)	Mean (SD)	Mean (SD)
**Age and body composition**			
Age (y)	26.3 (5.9)	24.8 (6.1)	28.7 (5.7)
Height (cm)	174.9 (7.8)	170.2 (5.7)	182.7 (1.5)
Weight (kg)	71.4 (8.9)	67.2 (9.0)	78.3 (1.1)
BMI (kg/m^2^)	23.4 (3.6)	23.4 (4.8)	23.5 (0.5)
Fat percent (%)	17.7 (8.5)	21.0 (9.4)	12.1 (1.0)
**Blood count measures**			
White blood cell count (/l)	4.7 × 10^9^ (1.5 × 10^9^)	5.3 × 10^9^ (1.6 × 10^9^)	3.7 × 10^9^ (0.7 × 10^9^)
Red blood cell count (/l)	4.5 × 10^12^ (0.4 × 10^12^)	4.3 × 10^12^ (0.3 × 10^12^)	4.9 × 10^12^ (0.4 × 10^12^)
Hemoglobin (g/l)	131.8 (13.1)	124.0 (5.8)	144.7 (11.5)
Hematocrit (l/l)	0.39 (0.03)	0.38 (0.02)	0.42 (0.02)
**Blood pressure and resting heart rate^#^**			
Systolic blood pressure (mm Hg)	120.3 (9.9)	119.6 (12.0)	122.0 (0.0)
Diastolic blood pressure (mm Hg)	70.4 (7.0)	71.6 (8.2)	67.5 (0.7)
Resting heart rate (bpm)	67.0 (7.6)	70.2 (5.8)	59.0 (5.7)
Training volume (h/week)*			
Total training volume	7.3 (3.8)	6.9 (2.7)	8.2 (7.0)
Training at intensity level 1	0.7 (0.7)	1.0 (0.7)	0.1 (0.0)
Training at intensity level 2	3.2 (3.0)	2.4 (1.2)	4.9 (5.6)
Training at intensity level 3	2.6 (1.5)	2.7 (1.7)	2.4 (1.1)
Training at intensity level 4	0.7 (0.3)	0.6 (0.3)	0.8 (0.3)
Training at intensity level 5	0.2 (0.2)	0.3 (0.2)	0.0 (0.0)

^#^Not recorded from one male subject. *Training volume available from 4 women and 2 men. Intensity level 1 = very light physical activity; level 2 = light aerobic training; level 3 = moderate intensity aerobic training; level 4 = training near anaerobic threshold; level 5 = maximum intensity training or competition.

### Experimental Design

After being accepted to the study, subjects entered a ∼4-week familiarization period. During this self-monitored period, subjects were advised to perform a 1 h cycling session twice a week to become accustomed to cycling (Ergoselect 200, Ergoline GmbH, Bitz, Germany). The mean familiarization period duration was 36 ± 18 days. The duration was considerably longer (71 days) in one subject due to required recovery from Achilles tendinopathy symptoms and shorter in one subject (11 days) due to scheduling issues. All the experimental tests were performed at standardized conditions. Alcohol and coffee intake, medications, sauna bathing, and vigorous exercise training were prohibited 24 h before each experiment. Due to sweat collection, subjects were advised to shower only with water and not to use personal hygiene products during the same 24 h period before experiments. On each morning of testing, a 330 ml/220 kcal Naturdiet banana-strawberry smoothie breakfast (Midsona Finland OY, Vantaa, Finland) was served 2–3 h before the test. Water intake (*ad libitum*) was allowed during the endurance experiments. Immediately prior to each experiment, subjects showered with water and brushed themselves with a bath brush to remove dust and other particles from the skin.

**FIGURE 1:**
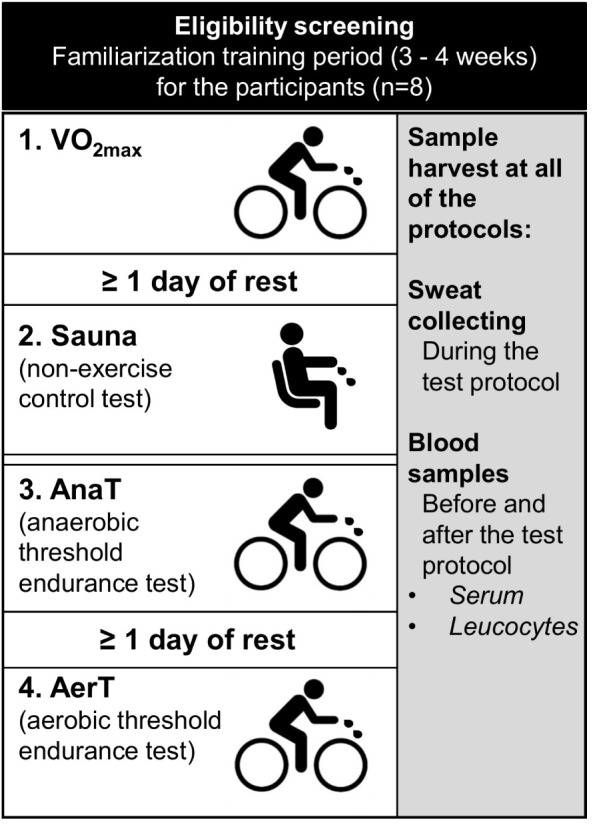
Schematic illustration of the study setup. After eligibility screening subjects (*n* = 8) went through a familiarization period followed by following tests in the same order: (1) VO_2__*max*_ test, (2) Sauna, (3) anaerobic threshold endurance test (AnaT), and (4) aerobic threshold endurance test (AerT). Same sample set was harvested in each of the tests.

The experiments included three endurance exercise tests performed with an electrically braked bicycle ergometer and a non-exercise control test (Sauna) (Figure 1). A minimum of one recovery day was scheduled between each test, except for the control measurement and anaerobic threshold test that were executed on subsequent days. During the recovery days, vigorous exercising was prohibited. On average, the measurements were executed within 19 days (±8.3, range 14–38) from the first experiment and always in the same order: (1) maximal aerobic capacity test (VO_2__*max*_), (2) control experiment (Sauna), (3) anaerobic threshold endurance test (AnaT), and (4) aerobic endurance test (AerT).

#### Maximal Aerobic Capacity Test (VO_2__*max*_)

A graded maximal exercise test was carried out to determine maximal oxygen uptake as well as aerobic and anaerobic thresholds for the subsequent exercise tests. A standardized 10-min warm-up at an exercise intensity of 30–70 W for women and 50–90 W for men preceded the test. For women, the test began at 70 W and for men, the first intensity was 90 W. The load was increased every 3 min by 20 W in women and by 30 W in men. The VO_2__*max*_ protocol was designed to allow determination of VO_2__*peak*_ as well as aerobic and anaerobic thresholds while lasting a sufficient duration to ensure adequate sweat secretion for sweat sample harvesting. The subjects were asked to maintain a pedaling frequency of 60 rpm throughout the test, which was monitored continuously. Subjects were encouraged to continue cycling until volitional exhaustion, and the test was terminated when the subjects failed to maintain the required cadence for more than 15 s. Breath-by-breath gas exchange was recorded with a Vmax Encore 29 metabolic cart (VIASYS Respiratory Care Inc., Palm Springs, CA, United States), which was calibrated according to the manufacturer’s instructions before each measurement. Maximal oxygen uptake (VO_2__*max*_) was determined as the highest VO_2_ averaged over 60 s (Mikkola et al., 2007). Maximal aerobic cycling power (Pmax) was calculated as Pmax = Pcom + (t/180)ΔP in which Pcom is the last cycling load completed, t is the time in seconds the non-completed load was maintained, and ΔP is the increment in watts (Kuipers et al., 1985). Heart rate (HR) was monitored throughout the test (Polar V800, Polar Electro Ltd., Kempele, Finland), and the 30 s average (from 2:15 to 2:45) of each stage was recorded. Capillary blood samples (20 μl) were collected from the fingertip into reaction capsules containing a hemolyzing and anticoagulant agent before the test, at the end of each stage, as well as immediately and 5 min after the test to determine blood lactate concentrations. Lactate concentrations were analyzed using a Biosen analyzer (C-line Clinic, EKF, Magdeburg, Germany). Subjects’ individual aerobic and anaerobic thresholds were determined using deflection points obtained by plotting the curves of blood lactate concentrations, ventilation, oxygen uptake, and carbon dioxide output (Aunola and Rusko, 1986).

#### Non-exercise Control Test (Sauna)

The control condition was a non-exercising test. Subjects rested in a seated position in the sauna (∼60°C) for a maximum of 30 min or when sweat collection was sufficient (seven subjects spent 25 min and 1 subject 30 min in sauna). Real-time lactate measurements were taken from the fingertip before, immediately after, and 5 min after the experiment (Lactate Scout, SensLab GmbH, Germany). HR was measured continuously, and rating of perceived exertion (RPE, Borg scale, 6–20) was recorded at 5-min intervals.

#### Anaerobic Threshold Endurance Test (AnaT)

Subjects cycled at a load corresponding to ∼anaerobic threshold determined in the VO_2__*max*_ test for 30 min. A 10-min warm-up period at an intensity of 15% below the aerobic threshold preceded the exercise. The subjects were asked to maintain a pedaling frequency of 60 rpm throughout the exercise, which was monitored continuously. RPE, HR, and blood lactate concentrations were determined at 5-min intervals. The load was adjusted during the exercise according to the responses in heart rate and real-time blood lactate concentrations (Lactate Scout), if necessary, to maintain physiological strain at a level of 5% below the anaerobic threshold. Lactate was also measured 5 min after the test.

#### Aerobic Threshold Endurance Test (AerT)

The final exercise protocol was 50 min cycling performed at a load corresponding to the aerobic threshold intensity. The total workload was designed to match the AnaT. A 10 min warm-up period at an exercise intensity of 15% below the aerobic threshold preceded the exercise. The subjects were asked to maintain a pedaling frequency of 60 rpm throughout the exercise, which was monitored continuously. RPE, HR, and real-time blood lactate concentrations (Lactate Scout) were determined at 10 min intervals, and lactate also 5 min after the exercise. The load was adjusted, during the exercise according to the responses in heart rate and blood lactate concentrations, if necessary, to maintain physiological strain at the level of aerobic threshold.

### Sweat Collection

Sweat was collected during the entire duration of each test with thin polyethylene veterinary gloves (VETbasic, Albert Kerbl GmbH, Buchbach, Germany) covering the whole left arm and right forearm and hand. As described in the section “Experimental Design,” each subject showered in water before the experiments and brushed themselves with a bath brush to remove dust and other particles from the skin. The right arm was used for blood sampling before the test experiment. The fingertip used for lactate measurement was left uncovered. Gloves were removed immediately after each test, sweat was collected and the total amount of sweat recorded. Sweat used for RNA isolation was purified with a 40 μm filter followed by a 0.8 μm filter to remove any skin, hair, or cell debris before being snap-frozen in liquid nitrogen. The remaining portion of sweat was frozen in liquid nitrogen and stored at −80°C for EV-isolation. Since sweat collection was the primary goal in the experiments, subjects were instructed to wear additional clothing to increase perspiration during AerT and AnaT. For the VO_2__*max*_ test, light clothing was advised so that the body temperature rise would not hinder test performance. The subjects wore a swimsuit during the Sauna test.

### Blood Sample Collection

Venous blood samples were collected at the eligibility screening visit and during each experimental test protocol from the antecubital vein in a supine position. At the eligibility screening visit, samples were taken following an overnight fast between 08:00 and 10:00 AM. Blood count, including a measure of hemoglobin and hematocrit (Sysmex KX-21N, Sysmex Corp., Japan), were obtained from whole blood. During each test, blood samples were collected immediately before and 10 min after the test. Separate samples were collected into PAXgene tubes (Qiagen, 762165, Hilden, Germany), to allow the isolation of RNA from leukocytes, and to serum-separator tubes. The serum sample was used to isolate EV and non-EV fractions of blood. For serum separation, the whole blood sample was allowed to clot for 30 min at room temperature and centrifuged at 2200 × *g* before aliquoting and storing sera at −80°C until analysis. In addition to venous blood samples, fingertip lactate measurements were taken at test-specific intervals.

### Validating EV Isolation

Before beginning the EV isolations from serum and sweat, test isolations were performed to validate the successful isolation of EVs. Isolations were made with ExoRNeasy Serum/Plasma Midi Kit (77044, Qiagen) from 500 μl of representative serum and 1 ml of representative pre-filtered sweat. Affinity-based columns were chosen for this study due to the relatively small volume of starting material needed and the capability of columns to remove the majority of miR-carrier proteins (AGO2) from serum samples (Enderle et al., 2015). Extracellular vesicles were eluted from the columns with 140 μl of elution buffer (XB, buffer, Qiagen), incubated for 5 min and centrifuged for 5 min at 500 × *g* at room temperature (RT) to collect the eluted EVs. Thereafter, 360 μl of filtered (0.2 μm filter) phosphate-buffered saline solution (PBS, pH 7.4) was mixed together with the EV fraction. The sample was then transferred to an ultra-filter device (Amicon, Millipore, Darmstadt, Germany) and centrifuged for 10 min at 14,000 *g* at RT. Flow through was discarded and the ultra-filter with EVs was washed twice with 450 μl of filtered PBS. The concentrate was recovered according to the manufacturer’s instructions. Samples were sent to the University of Helsinki EV Core (Helsinki, Finland) for Nano-Particle tracking (NTA) and electron microscope (EM) analyses.

Extracellular vesicles were prepared for EM as described earlier (Puhka et al., 2017). Briefly, EV samples were loaded on 200 mesh grids, fixed with 2% paraformaldehyde solution (PFA), stained with 2% neutral uranyl acetate and embedded in uranyl acetate and methyl cellulose mixture (1.8/0.4%). Extracellular vesicles were viewed with transmission EM using Jeol JEM-1400 (Jeol Ltd., Tokyo, Japan) operating at 80 kV. Images were taken with Gatan Orius SC 1000B CCD-camera (Gatan Inc., United States).

Purified EV samples were analyzed by NTA using Nanosight model LM14 (Nanosight) equipped with blue (405 nm, 60 mW) laser and SCMOS camera. The samples were diluted in 0.1 μm filtered (Millex VV, Millipore) Dulbecco’s PBS (DPBS) to obtain 40–100 particles/view, and five 30 s videos were recorded using camera level 13 with automatic temperature setting of 22°C. The data were analyzed using NTA software 3.0 with the detection threshold 5 and screen gain at 10 to track as many particles as possible with minimal background.

### EV and RNA Isolation From Serum and Sweat

Serum samples were first centrifuged for 10 min at 16,000 *g* (Heraeus, Biofuge Pico, ThermoFisher, United States). ExoRNeasy Serum/Plasma Midi Kit (77044, Qiagen) was used to isolate EVs and RNA from 0.5 ml of serum according to the manufacturer’s instructions. Extracellular vesicles and RNA from sweat samples were isolated from 1 ml of pre-filtered sweat sample with exoRNeasy Serum/Plasma Midi Kit (77044, Qiagen) according to the manufacturer’s instructions. After binding the EVs into the affinity-based column, 1 ml of the filtrate that passed through the membrane was collected to study the non-EV fraction of the samples. The RNA was isolated from 200 μl of the filtrate using miRNeasy Serum/Plasma Mini Kit (217004, Qiagen) according to the manufacturer’s instructions. For all the samples except for PAXgene, miR-39 spike-in control was used.

### RNA Isolation From Leukocytes

PAXgene tubes were used to isolate RNA from leukocytes. RNA isolation was done with PAXgene Blood miRNA Kit (763134, Qiagen) according to the manufacturer’s instructions. Briefly, PAXgene Blood RNA tubes were first centrifuged for 10 min at 5,000 *g* using a swing-out rotor (Heraeus Megafuge 16R, Thermo Fisher Scientific Inc., Waltham, MA, United States). During the isolation protocol a rotational speed of 5,000 *g* was used to centrifuge the tubes whenever a range of 3,000–5,000 *g* was recommended. After pipetting the samples into PAXgene Shredder spin columns, samples were centrifuged for 3 min at 16,000 *g* (Heraeus Biofuge Pico, Thermo Fisher Scientific Inc). The highest speed of the Biofuge Pico centrifuge was 16,000 *g*, and this was used whenever range of 8,000–20,000 *g* was recommended in the protocol. The final elution of the RNA sample was done with 2 × 30 μl of buffer BR5. After the isolation, the concentration and purity of RNA were measured (NanoDrop) and samples were immediately stored at −80°C.

### cDNA Synthesis and qPCR From PAXgene, Serum and Sweat Samples

After RNA isolation, cDNA synthesis was performed with miScript II RT Kit (218161, Qiagen) according to the manufacturer’s instructions. From PAXgene samples, 500 ng of RNA was used for the cDNA synthesis and 12 μl of isolated RNA was used from serum and sweat EV and non-EV fractions. For the qPCR runs, 4 ng of PAXgene sample was used in each well, and 1 μl of non-diluted cDNA was used from serum and sweat. Samples were run as duplicates (serum and sweat) or triplicates (PAXgene). As a housekeeping gene, RNU6 (MS00033740, Qiagen) was used for PAXgene samples, and spike-in control cel-miR-39-3p (MS00019789, Qiagen) for serum and sweat samples. The studied miRs were miR-21-5p (MS00009079, Qiagen), miR-26a-5p (MS00029239, Qiagen), miR-126-3p (MS00003430, Qiagen) miR-146a-5p (MS00001638, Qiagen), miR-221-3p (MS00003857, Qiagen), and miR-222-3p (MS00007609, Qiagen). qPCR protocol was the following: 95°C (15 min, activation), 94°C (15 s), 55°C (30 s) and 70°C (30 s) with 40 cycles. Relative expression of the studied miRNAs was calculated by using equation 2^–Δ^ ^*Cq*^, where ΔCq = target miR Cq-value – housekeeping Cq-value. For sweat miRs, the results were expressed as fold change relative to the Sauna level.

### Western Blot Analyses

Western blot (WB) analysis was carried out to explore the possible differences in the content of sweat and serum EV fractions. For this purpose, three commonly used EV antibodies (CD63, CD9, and TGS101), miR-binding protein argonaute 2 (AGO2) antibody, and HDL-derived protein (APOA1) antibody were used. EVs from a subset of the sweat and serum (post-exercise/sauna) samples (*n* = 4) were first isolated as described in validating EV isolation with the exception that 1 ml of sweat was used as a starting material. After harvesting the concentrate, the samples were pooled together forming eight different EV pools: sweat: Sauna, VO_2__*max*_, AnaT, AerT, and serum (post-exercise/sauna): Sauna, VO_2__*max*_, AnaT, and AerT. These pooled samples were then used for the WB analysis. Pooled samples were solubilized in Laemmli sample buffer and heated at 95°C for 10 min to denature proteins and separated by SDS-PAGE for 60–90 min at 270 V using 4–20% gradient gels on Criterion electrophoresis cell (Bio-Rad Laboratories, Richmond, CA). Proteins were transferred to PVDF membranes in a Turbo blotter (Trans-Blot Turbo Blotting System, 170-4155, Bio-Rad Laboratories). The homogeneity of protein loading of the serum samples was checked by staining the membrane with Ponceau S. Membranes were blocked in commercial blocking buffer [Odysseu Blocking Buffer (PBS), Licor] for 2 h and then incubated overnight at 4°C with commercially available primary antibodies to measure the following protein contents with stated dilutions: CD63 (1:500; ab59479, Abcam, Cambridge, United Kingdom), CD9 (1:1,000; ab92726, Abcam), TGS101 (1:1,000; ab125011, Abcam), AGO2 (1:1,000; ab32381, Abcam), and APOA1 (1:10,000; ab52945, Abcam). Afterwards, the primary antibody incubation membranes were washed in TBS-T, incubated with suitable secondary antibody (1: 10,000), and diluted in 1:1 Pierce blocking buffer and TBS-T for 1 h followed by washing in TBS-T. Proteins were visualized by fluorescence using ChemiDoc XRS in combination with Quantity One software (version 4.6.3. Bio-Rad Laboratories).

### Statistical Analysis

Data are presented as means and standard deviations (SD) in tables and as means + standard error of means (SEM) in the figures. The normality of the variables was assessed with the Shapiro–Wilk test. As only a few of the parameters met the normal distribution criteria, the analyses were run using non-parametric tests. First, the extreme outliers were excluded from the analysis [>3× interquartile range (IQR)]. Statistical analysis of sweat qPCR results was run first with Friedman’s test to verify whether there were differences between the exercise protocols. When a significant difference was found (*p* ≤ 0.050), a Wilcoxon test was used as a *post hoc* test. Serum and leukocyte pre- and post-exercise/sauna samples were analyzed with Wilcoxon’s test. Correlation analyses between sweat and serum (post-exercise/sauna) miR expression levels from EV fraction and sweat volume and sweat miR expression level from EV fraction were run using Spearman’s test. Data analysis was carried out using IBM SPSS Statistics software version 24 (Chicago, IL, United States), and the level of significance was set at *p* ≤ 0.050.

## Results

### Study Subjects

The characteristics of the study subjects (*n* = 8) are presented in Table 1. All the study subjects were healthy and performed exercise on a regular basis.

### Endurance Exercise Tests

The key characteristics of the Sauna and endurance exercise tests are presented in Table 2. The heart rates and lactate levels are in line with the different intensities of the test protocols. Collected sweat volumes show variation between tests as well as between subjects.

**TABLE 2:** Results of maximal aerobic capacity test and threshold determination.

	All (*n* = 8)	Women (*n* = 5)	Men (*n* = 3)
	Mean (SD)	Mean (SD)	Mean (SD)
**Maximal aerobic capacity test (VO_2__*max*_)**			
VO_2__*max*_ (ml/kg/min)	49 (6)	45 (4)	56 (3)
RER	1.15 (0.05)	1.13 (0.05)	1.17 (0.03)
HR_*max*_ (bpm)	194 (7)	190 (6)	198 (6)
HR_*max*_/APMR (%)	100 (4)	97 (2)	104 (2)
W_*max*_ (W)	242 (46)	212 (22)	292 (15)
Lactate (mmol/l)	11.4 (3.5)	9.5 (1.7)	14.6 (3.6)
**Anaerobic threshold determination**			
VO_2_ (ml/kg/min)	38 (5)	35 (3)	42 (3)
%VO_2__*max*_ (%)	77 (4)	76 (3)	78 (4)
HR (bpm)	176 (6)	176 (8)	176 (6)
W (W)	186 (40)	160 (10)	230 (26)
Lactate (mmol/l)	3.7 (0.8)	3.6 (0.6)	3.9 (1.1)
**Aerobic threshold determination**			
VO2 (ml/kg/min)	27 (5)	25 (3)	32 (4)
%VO_2__*max*_ (%)	55 (4)	54 (4)	57 (1)
HR (bpm)	145 (10)	147 (13)	143 (3)
W (W)	131 (30)	111 (13)	165 (9)
Lactate (mmol/l)	1.4 (0.2)	1.4 (0.2)	1.3 (0.2)

*VO_2_, oxygen uptake, HR, heart rate; RER, respiratory exchange ratio; W, workload; APMR, age-predicted maximal heart rate (220-age).*

The detailed results of VO_2__*max*_ and threshold determination are presented in Table 3. Based on the RER, HR, lactate, and RPE measurements, the subjects reached maximal exertion level. The exercise intensities reached in AnaT and AerT (Table 2) corresponded well with the predetermined threshold levels, confirming that the exercise tests were performed as planned.

**TABLE 3:** Key test characteristics during test protocols.

	Sauna	VO_2__*max*_	AnaT	AerT
	Mean (SD)	Mean (SD)	Mean (SD)	Mean (SD)
**Test characteristics**				
Duration (min)	26 (2)	26 (3)	30	50
Mean HR (bpm)	86 (6)	194(7)^§^	170 (6)	148 (8)
Mean W (W)		242(46)^§^	181 (43)	136 (34)
Mean RPE (6–20)	7 (1)	19(1)^§^	15 (1)	12 (1)
**Lactate (mmol/l)**				
Before test/warm-up	1.1 (0.4)	1.2 (0.3)	1.1 (0.3)	1.0 (0.5)
After warm-up		1.1	1.1 (0.3)	1.1 (0.5)
Mean during test			4.0 (1.2)	1.7 (0.5)
Peak during test		11.4(3.5)^§^	4.9 (1.3)	2.6 (2.1)
5 min after	0.8 (0.3)	9.9 (1.7)	3.5 (1.0)	1.2 (0.3)
**Change in body composition**				
Body mass (kg)	−0.33(0.23)	−0.48(0.25)	−0.65(0.24)	−0.69(0.36)
Body water (l)	0.55(0.46)	−0.11(0.42)	0.30 (0.76)	0.15 (0.58)
Intracellular water (l)	0.38 (0.43)	−0.13(0.29)	0.13 (0.48)	0.09 (0.35)
Extracellular water (l)	0.26 (0.18)	0.01 (0.16)	0.18 (0.30)	0.06 (0.26)
*Collected sweat (ml)*	22 (8)	35 (27)	61 (24)	88 (48)

*^§^Based on maximal values during the test. HR, heart rate; W, workload; RPE, rating of perceived exertion.*

### Validation of Sweat and Serum EV Isolation

Successful EV isolations from serum and sweat EVs were confirmed with NTA and EM analyses from representative samples (Supplementary Figure 1). NTA analysis showed particles at the size range 100–150 nm and EM figures show EVs.

**FIGURE 2:**
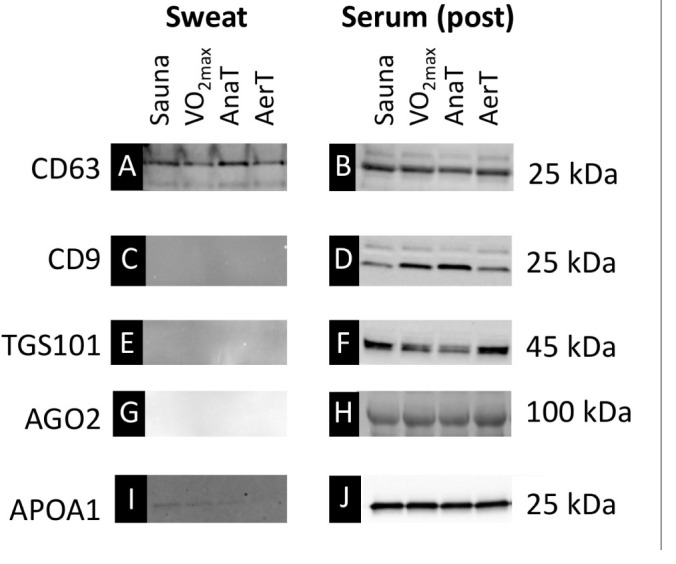
Western blot (WB) images from pooled (*n* = 4/group) EVs isolated from sweat and serum (post-samples). EV marker CD63 was detected both from sweat **(A)** and serum **(B)** samples, whereas EV markers CD9 and TGS101 were detected only from serum samples (**D,F** vs. **C,E**). Similarly, AGO2 was only present in serum samples (**G** vs **H**). HDL marker APOA1 was clearly present in serum samples **(J)** and showed weak positive signal also from some of the sweat samples **(I)**.

Western blot analysis was carried out from pooled samples (*n* = 4) of serum (post-exercise/sauna) EV and sweat EV fraction from all the studied test protocols (Figure 2). Both sweat and serum EV samples showed positive signals with CD63 EV antibody (Figures 2A,B), whereas only serum showed a positive signal with CD9 and TGS101 EV antibodies (Figures 2D,F). AGO2, that carries protein-bound miRs, had a positive signal only in the serum sample (Figures 2G,H), whereas HDL-associated APOA1 showed a strong positive signal from all serum samples but only a very weak signal from part of the sweat samples (Figures 2I,J).

### miR Analyses From Sweat and Serum EV Fraction

#### Sweat

**FIGURE 3:**
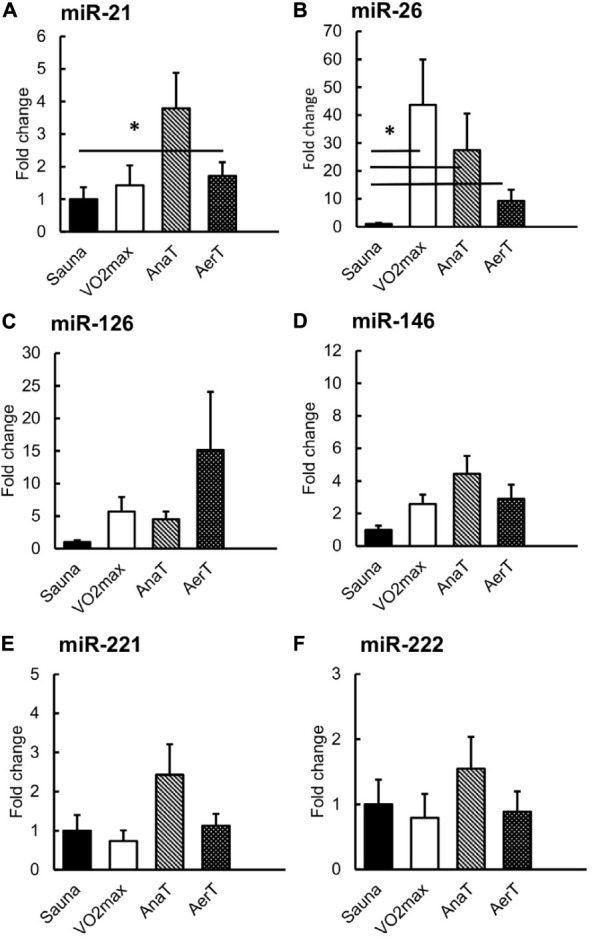
miR expression in sweat EVs in control (Sauna) or endurance exercise tests (*n* = 6–8). The expression of miR-21 was increased after AerT **(A)**, whereas miR-26 was increased after VO_2__*max*_, AnaT and AerT tests compared with sauna **(B)**. There were no differences in miRs -126 **(C)**, -146 **(D)**, -221 **(E)** or -221 **(F)**. *p ≤ 0.050. Figure shows mean + SEM.

Friedman’s test showed significant difference in miR-21, -26, and -221 (*p* = 0.020, *p* = 0.004, and *p* = 0.018, respectively). Compared to Sauna value, miR-21 and miR-26 expressions increased in sweat EVs after the AerT (*p* ≤ 0.050, Figures 3A,B). MiR-26 was also increased in sweat EVs after VO_2__*max*_ and AnaT tests compared with Sauna (*p* = 0.028, Figure 3B).

#### Serum

**FIGURE 4:**
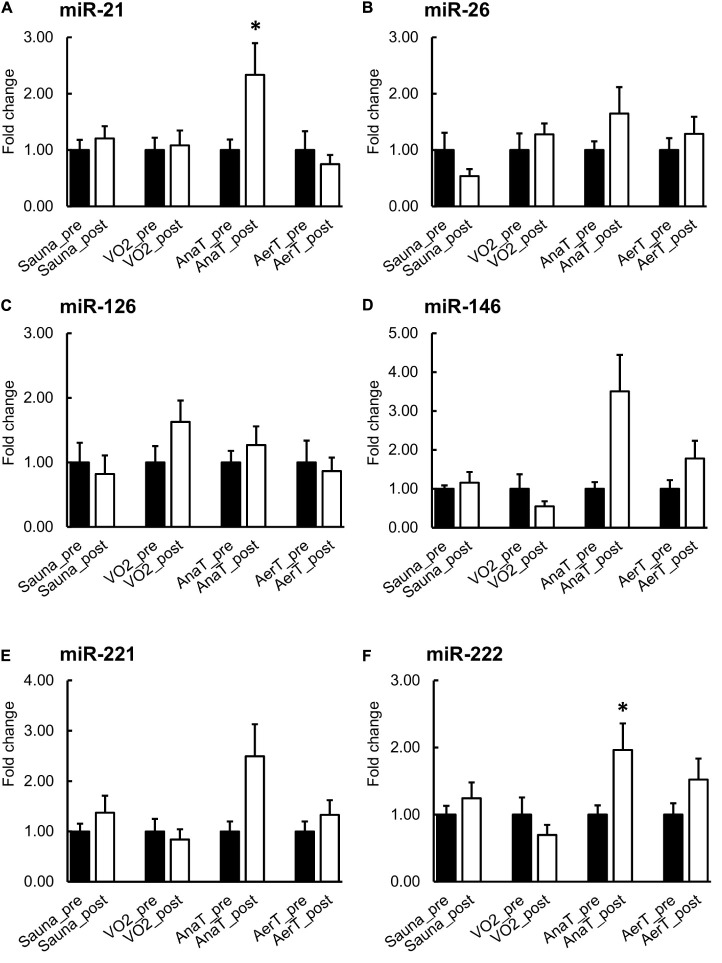
miR expression in serum EVs before and after control (Sauna) or endurance exercise tests (*n* = 7–8). The expression of miR-21 and miR-222 were increased after AnaT **(A,F)**. There were no changes in the rest of the studied miRs in any of the exercise tests **(B–E)**. *p ≤ 0.050. Figure shows mean + SEM.

When comparing serum pre- and post-expression levels, miR-21 and miR-222 levels increased in serum EVs after AnaT and there was a trend for increased miR-146 (*p* = 0.063, Figure 4C). There was also a trend for decrease in miR-26 after the Sauna (*p* = 0.063, Figure 4B). No significant changes were observed following Sauna or any exercise test (Figure 4). In Supplementary Figure 2 serum results are presented for each test in arbitrary units (AU), showing the variation in the expression levels of the studied miRs.

#### Correlation Analysis of miR-Expression

Table 4 shows correlation analyses of the studied miRs from sweat and serum EVs. There was a significant positive correlation in miR-146 in AnaT (*r* = 0.881, *p* = 0.004), but there were no consistent positive correlations between other studied miRs in other exercise tests. However, there was a negative correlation in miR-26 in Sauna (*r* = −0.714, *p* = 0.047). In addition, there was a trend toward a negative correlation in miR-146 expression in VO_2__*max*_ and toward a positive correlation in miR-21 in AnaT (*r* = −0.667, *r* = 0.667, respectively; *p* = 0.071 for both). Supplementary Table 1 shows the correlation between sweat volume and the studied miRs from sweat EVs. No significant correlations were observed in any of the studied miRs in the exercise tests used in this study.

**TABLE 4:** Correlation analysis of sweat and serum (post-sample) miR expression levels from EV fraction.

	Target	*r*	*p*
Sauna	miR-21	–0.119	0.779
	miR-26	–0.714	**0.047**
	miR-126	–0.548	0.160
	miR-146	0.143	0.736
	miR-221	0.190	0.651
	miR-222	0.310	0.456
VO_2__*max*_	miR-21	0.214	0.610
	miR-26	–0.357	0.385
	miR-126	–0.333	0.420
	miR-146	–0.667	0.071
	miR-221	0.333	0.420
	miR-222	–0.310	0.456
AnaT	miR-21	0.667	0.071
	miR-26	0.619	0.102
	miR-126	0.190	0.651
	miR-146	0.881	**0.004**
	miR-221	0.643	0.086
	miR-222	0.619	0.102
AerT	miR-21	0.071	0.867
	miR-26	0.048	0.911
	miR-126	–0.571	0.139
	miR-146	–0.333	0.420
	miR-221	0.405	0.320
	miR-222	–0.167	0.693

*Statistically significant (*p* ≤ 0.050) values are bolded.*

### Leukocytes

Supplementary Figure 3 shows results from blood leukocytes. In leukocytes, miR-126 and miR-221 expression increased after Sauna (*p* ≤ 0.050) and miR-21 and miR-126 after VO_2__*max*_ (*p* ≤ 0.050) compared with the corresponding pre-value. There were no changes in the miR levels in other studied exercise tests.

### miR Analyses From Non-EV Fraction

#### Sweat

In addition to sweat EVs, all studied miRs were also detected in sweat non-EV fractions, but their expression pattern across different tests was mostly not in line with EV fraction results. In sweat non-EV fraction, Friedman’s test showed significant difference in miR-21, -26 and -146 (*p* = 0.047, *p* = 0.044, and *p* = 0.019, respectively). There was an increase in miR-26 and miR-146 levels after VO_2__*max*_ and in miR-26 after AnaT compared with Sauna (Supplementary Figure 4, *p* < 0.050). There were no significant changes in the other studied miR expression levels in the non-EV fraction of sweat compared with Sauna. Of the miRs studied here, only miR-26 patterns followed similar trends in EV (Figure 4B) and non-EV fractions.

#### Serum

There were no significant changes in the miR expression in the non-EV fraction of serum when comparing the pre- and post-levels (Supplementary Figure 5). In general, the miR-levels in the non-EV fraction of serum followed similar pattern as seen in the EV fractions, yet there were differences in which miRs enrich into the EV fraction and which into the non-EV fraction.

## Discussion

The results of our preliminary study revealed that sweat holds a distinct miR carrier protein content compared with serum samples harvested from the same study subjects. In addition, our study is the first to show that sweat EVs carry miRs and that the level of two of these miRs alter in response to different exercise intensities compared with non-exercise control (Sauna).

### EV Protein Marker CD63 Enrich in Sweat

With the affinity-based purification of EVs used here, serum-derived EVs contained all the tested EV protein markers (CD63, CD9, and TGS101) indicating the presence of a broad variety of EV populations, while only CD63 protein containing EVs were found in sweat (Figure 2). The first paper to show that EVs are present in sweat by Wu and Liu (2018) had similar findings; they revealed that EV markers HSP70 and ALIX were present in both sweat EVs and 293T cells, whereas CD63 marker was only detectable in sweat EVs (Wu and Liu, 2018). Our study is the first to indicate that with the same study subjects, CD63 EV-marker protein enriches in sweat whereas several additional EV markers are detectable in serum. Previous studies indicate that all body fluids contain a heterogeneous mixture of EVs derived from various cells of the body (Yanez-Mo et al., 2015). Yet to date it has not been possible to identify certain EV populations based solely on the expression of EV-markers. It seems that skin acts as a selective barrier altering the EV content that enters skin surface via sweat glands. This should be taken into consideration when planning analyses from sweat as a substitute for serum.

### miRs Enrich to Sweat EVs

In contrast to EV protein markers, we were able to detect all the chosen miRs (miR-21, -26, -126, -146, -221, and -222) in sweat EVs (Figure 3). Our results also showed that miR-response to endurance exercise tests was most prominent in the EV fraction of serum and sweat (showing significant changes), as we analyzed both the EV (Figures 3, 4) and non-EV (Supplementary Figures 4, 5) fractions’ miR content from the same samples. Our analyses revealed that the miR content in non-EV fraction is not similar with the miR content of EV fraction of serum or sweat, indicating that miRs are selectively packed into EVs whereas non-EV fraction may contain miRs after more random release from the cells. In sweat, the miR-26 pattern in non-EV fraction followed a similar trend as in EVs, yet the increase was not significant in the non-EV fraction (Supplementary Figure 4). In serum, miRs-21 and -222 were enriched in EVs after AnaT whereas there was no change in the non-EV fraction (Figure 4 and Supplementary Figure 5).

In a previous study, Harshman et al. collected sweat after treadmill running and analyzed it for metabolite and protein content (Harshman et al., 2018). However, due to the very low protein content in sweat, they stated that significant enrichment steps would be necessary for proteomic biomarker discovery from a single sweat sample. Wu and Liu (2018) made an attempt to overcome this issue by isolating sweat EVs and found that proteins enrich in sweat EVs (Wu and Liu, 2018). Our results support the finding that, in addition to proteins, miRs are actively selected and enriched to sweat EVs and are not just passively circulating in any fraction of the blood or sweat.

### miRs-21 and -26 Carried in Sweat EVs Respond to Different Exercise Intensities

Previous studies have found several c-miRs in serum or plasma, that respond to acute exercise stimuli (for a review, see Sapp et al., 2017). Our study is the first to show that in addition to serum, also sweat EV-carried miRs respond differently to endurance exercise tests with distinct intensities (Figure 3). Of the miRs studied here, miR-21 increased specifically in response to the low-intensity AerT test. Interestingly, EV-carried miR-26 showed a stepwise increase as the exercise loading intensified (AerT – AnaT – VO_2__*max*_). The increase in miR-26 level was drastic >40-fold after VO_2__*max*_, almost 30-fold after AnaT, and 10-fold after AerT in sweat EVs (Figure 3B). Analysis from serum EV miRs revealed that body temperature elevation triggered by Sauna did not alter the level of the studied miRs (Figure 4). Hence, our findings support that the previously observed systemic miR responses to endurance exercise are triggered by exercise, and not elevated body temperature *per se*. In serum EVs, only AnaT induced a change in the studied miRs, where the levels of miRs-21 and -222 were increased (Figures 4A,F).

A previous exercise study from Baggish et al. (2011) showed that miRs-21 and -222 respond acutely to VO_2__*max*_ test (performed by bicycle ergometer) by increased expression in plasma immediately after exercise and that the level decreases 1 h after training in healthy men (Baggish et al., 2011). However, in the present study VO_2__*max*_ test did not change miR-levels (Figure 4). There are several possible explanations for the different results observed regarding miR-21 levels. The study by Baggish et al. (2011) used male subjects who were younger and had better overall fitness than the subjects of our study. Also, the sample processing, total RNA isolation and miR quantification methods may affect levels of c-miRs. Most importantly, Baggish et al. (2011) studied c-miRs from plasma, including all c-miR carriers, whereas we focused on miRs specifically sorted to EVs and released to circulation and used serum as a source to isolate EVs.

miR-21 has been shown to consistently respond to hypoxia, and its promoter has a hypoxia-induced factor 1 (HIF-1)-binding site (Kulshreshtha et al., 2007). As for the exercise tests used in the present study, AnaT caused hypoxic conditions in contracting myofibers for an extended period of time. Thus, it is logical that this protocol also induced an increase in serum miR-21 expression. Interestingly, miR-21 in sweat increased in response to AerT test. Previously Wu and Liu (2018) suggested that sweat exosomes are involved in skin immunity whereas miR-21 has shown to be a key switch in anti-inflammatory responses (Sheedy, 2015; Wu and Liu, 2018). Our results may thus indicate that miR-21 is enriched in sweat after prolonged endurance exercise to promote anti-inflammatory response at the skin surface.

A previous study demonstrated that miR-222 regulates angiogenesis of endothelial cells via c-Kit expression (Poliseno et al., 2006). Hence, it is likely that also miR-222 in serum responds strongly to exercise where the muscles work closest to hypoxia, much like the case of miR-21. Indeed, miR-222 has been shown to be necessary for exercise-induced cardiac growth by promoting physiological hypertrophy (Liu et al., 2015). Conversely, miR-26 has been shown to be downregulated in plasma immediately after a marathon run (Clauss et al., 2016). These results suggest that in blood, miR-26 responds to extremely prolonged aerobic exercise but not to short term (30–50 min) endurance exercise bouts. miR-26 is known to regulate angiogenesis by targeting endothelial cell signaling (Icli et al., 2013). Interestingly, systemically delivered miR-26a mimics inhibited exercise-induced angiogenesis in mice impairing physiological angiogenic response. Based on these findings we speculate that miR-26 may enrich in sweat due to efficient transfer out from the working muscle cells to create a more favorable environment for exercise adaptations.

Our correlation analyses revealed that sweat EV miR content after control and endurance exercise tests correlated poorly with serum EV miR content (Table 4). There was a negative correlation in miR-26 in the Sauna and a positive correlation in miR-146 in AnaT. Overall, the correlations in miRs between sweat and serum EVs were weak. This observation may be explained due to the differences in EV carriers present in sweat and serum (Figure 2). The lack of correlation found in our study further indicates that sweat EVs are not simply sub-fractions of blood EVs released by sweat glands.

According to our findings, there is a possibility that leukocytes do not contribute to miR content in EVs of serum during exercise tests. Our results showed that the expression of miR-126 increased after Sauna and VO_2__*max*_ tests and miR-221 after Sauna in leukocytes. In serum EVs, we did not find similar responses, suggesting that blood leukocytes do not significantly contribute to the miR content of serum EVs. A previous study showed that leukocytes do contribute to the exercise-induced release of EVs into the circulation (Brahmer et al., 2019). Here, we did not examine the origin of EVs, yet our results indicate that the changes seen in serum EV miRs originate from other cells and tissues than blood leukocytes.

Skeletal muscle cells release various types of EVs (Rome et al., 2019), and exercise stimulus (e.g., marathon run) has previously been shown to significantly increase circulating muscle-specific miRs, −1 and −133a (Mooren et al., 2014), indicating active contribution of muscle tissue in c-miR levels after exercise. In addition, during exercise, mechanical stimuli such as sarcomere length changes during muscle contractions, skeletal muscle blood flow, and, therefore, shear stress and mechanical stretch in capillaries are elevated (Brown and Hudlicka, 2003). In the case of a selective c-miR export in serum EVs, it can be speculated that angiogenic miRs are released from endothelial cells into the circulation in response to hypoxia and/or shear stress (Wahl et al., 2016) in addition to the likely contribution of the actively contracting muscle cells. Another possible source of c-miRs are apoptotic or damaged cells caused by high-intensity exercise (Altana et al., 2015; Xu et al., 2015). However, as our study focused on miRs carried in serum EVs, the results of our study do not include apoptotic bodies, whereas it may explain some of the observations from studies using serum/plasma. Yet the potential differential stimuli and associated molecular responses between different exercise intensities/muscle work are still poorly understood and warrant more investigations. Even less is known about the effects of exercise on miRs in sweat EVs. Our study lays the groundwork for future studies investigating the exercise response and potential role of miRs in sweat.

### Study Strengths and Limitations

This preliminary study had exceptionally well-controlled exercise test protocols that were performed by the same study subjects in a repeated measures design. The subjects’ individual aerobic and anaerobic thresholds were determined by VO_2__*max*_ test and blood lactate concentrations. The well-controlled exercise protocols together with a novel non-exercise control test (Sauna) enabled us to draw more solid conclusions from our data than from previous literature. In our study, the miR-carrier vesicles were studied comprehensively via NTA, EM, and WB analysis, revealing the differences in serum and sweat miR-carriers. However, one should be aware of the detection limits of especially the WB method, as the EVs in our study were isolated from a small volume of sweat. We chose to use affinity-based columns in our present study. Other EV isolation methods include ultracentrifugation, which requires high volumes of (pooled) samples, and precipitation, which does not exclude non-EV miRs from serum (Karttunen et al., 2018). As the aim was to compare the serum and sweat EV-miR populations, affinity-based columns were the most promising tool for our setup (Enderle et al., 2015). However, our study revealed that besides EVs, serum samples contained also miR-carrying AGO2-protein and HDL-particles, even though AGO2 was clearly enriched in the non-EV fraction of serum (*data not shown*). Another limitation in our study is the small number of subjects, however, already with an n of eight, we were able to observe differences both in sweat and serum EV miR composition after the exercise tests. In addition, we were not able to measure the specific changes in body temperature during the test protocols, yet it is well-known that hot environment, such as sauna bathing, and vigorous exercise cause an elevation in body temperature initiating perspiration (Leppaluoto, 1988; Gleeson, 1998). A limiting factor is also the relatively low miR-expression levels, especially in the sweat samples. However, the duplicates run via qPCR showed excellent repeatability, suggesting that the miR-expression levels observed were reliable. One contributing factor to the variation in our results is the sweat volume, which was variable between the study subjects (Table 3). However, we found no correlation between sweat volume and miR level (Supplementary Table 1), suggesting that volume of sweat does not significantly contribute to miR expression level. Also, especially in serum EVs, the miR-levels at baseline varied between the test days despite controlling the conditions before (nutrition, sleep, and exercise) and during (standard conditions) the tests. The present preliminary study was a collaborative project to study sweat properties at RNA and protein levels. Hence, a large volume of sweat was needed to enable performing several analysis methods. For real-life online monitoring, the sweat collecting method used here is not optimal, but there is ongoing research on small adhesive bandage-type sweat collectors possibly combined with analytes (Corrie et al., 2015; Gao et al., 2016). Currently, the limiting step of using sweat as biomarker source for exercise monitoring is the methodology; there is a need to invent new technologies to robustly detect very small amounts of biomarkers. Also, studies with larger populations need to be carried out to examine the inter-individual variation in response to exercise in sweat. In the future, sweat-based analytics could be used in monitoring of body’s response to exercise parallel with other non-invasive monitoring systems, such as heart rate.

## Conclusion

Our preliminary study is the first to show that sweat EVs carry miRs and that certain miRs in sweat alter in response to different exercise intensities. Our results further revealed that non-EV fraction miR content did not reflect the miR content of serum and sweat EVs, which is in line with the idea of selective miR loading to EVs. Our findings from serum EVs support the previously found c-miR responses to endurance exercise are indeed triggered by exercise, and not due to increased body temperature. Based on our results sweat possesses a unique miR carrier content that should be taken into account when planning analyses from sweat as a substitute for serum.

## Data Availability Statement

All datasets generated for this study are included in the article/Supplementary Material.

## Ethics Statement

The study was approved by the local ethics committee (University of Jyväskylä, Finland). The patients/participants provided their written informed consent to participate in this study.

## Author Contributions

UK, JA, EL, SV, GB, and AS designed the study and analysis protocols. SK, JK, and TS did the statistical analysis and drafted the manuscript. PH, TS, and JK executed the exercise loading protocols and sample harvest. SK, TS, and JK did the biochemical analysis. All authors contributed to the revision of the manuscript and approved the final version of the manuscript.

## Conflict of Interest

The authors declare that the research was conducted in the absence of any commercial or financial relationships that could be construed as a potential conflict of interest.
