# Supplementary material for: MicroRNAs in Extracellular Vesicles in Sweat Change in Response to Endurance Exercise
Source: Front Physiol. 2020 Jul 15;11:676. doi: 10.3389/fphys.2020.00676 (PMC7373804; doi:10.3389/fphys.2020.00676)
Supplement: Supplementary file 1 [file Table_1.docx]

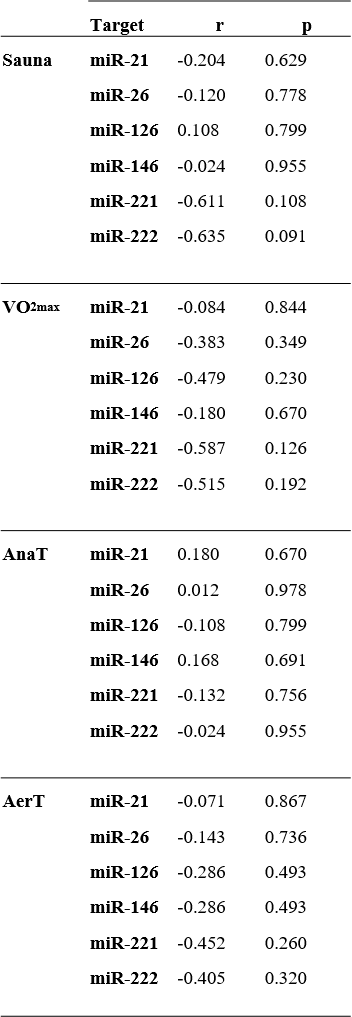


**Supplementary Table 1**. Correlation analysis of sweat volume (ml) and sweat miR expression from EV fraction**.**
