# Supplementary material for: MicroRNAs in Extracellular Vesicles in Sweat Change in Response to Endurance Exercise
Source: Front Physiol. 2020 Jul 15;11:676. doi: 10.3389/fphys.2020.00676 (PMC7373804; doi:10.3389/fphys.2020.00676)
Supplement: Supplementary file 2 [file Data_Sheet_1.docx]

Supplementary Material


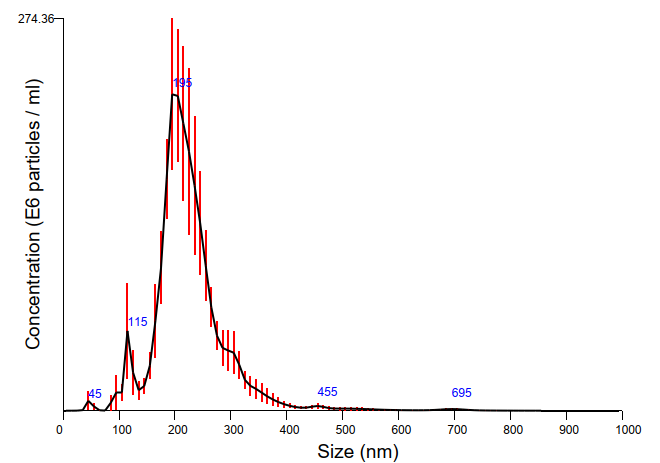

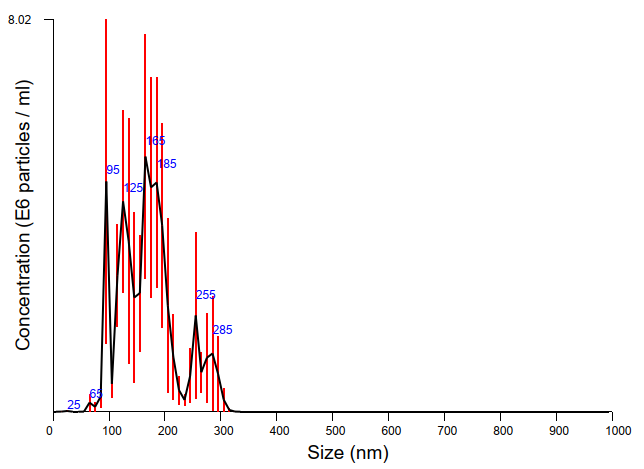


**A**

**B**

**C**

**D**


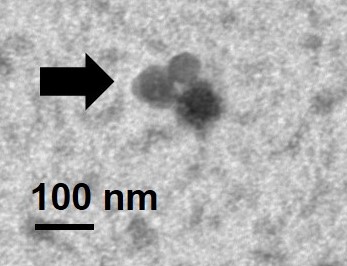

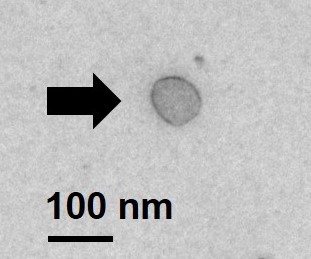


**Supplementary Figure 1.** NTA and EM analyses from EVs isolated from representative serum (A,B) and sweat (C,D) samples. Figures A and C show concentration of particles from NTA analysis with error bars in red (SEM). Figures B and D show corresponding EM image of the sample containing EVs. Black arrows point to EVs.

**
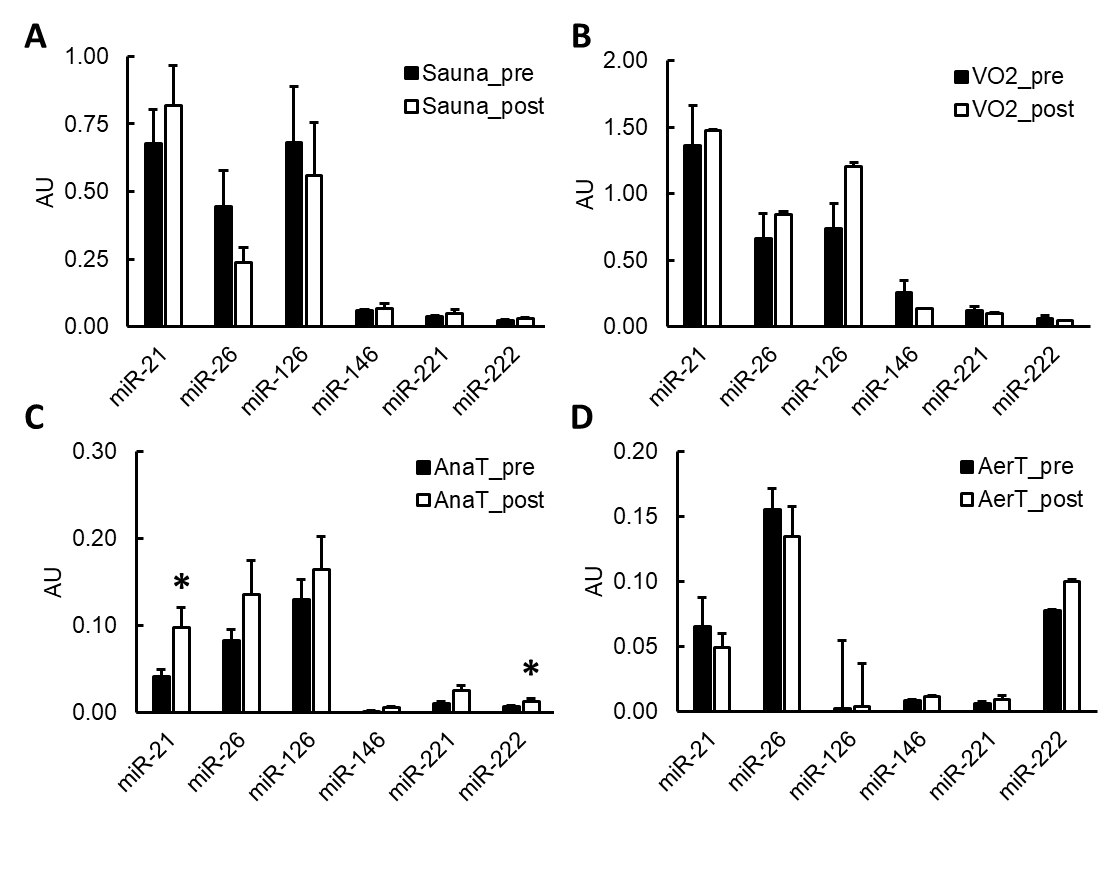
Supplementary Figure 2.** miR expression in serum EVs before and after control (sauna) or endurance exercise tests (n=7-8) in arbitrary units (AU). The expression of miR-21 and miR-222 were increased after AnaT (C). There were no changes in the rest of the studied miRs in any of the exercise tests. *p≤0.050. Figure shows mean+SEM.


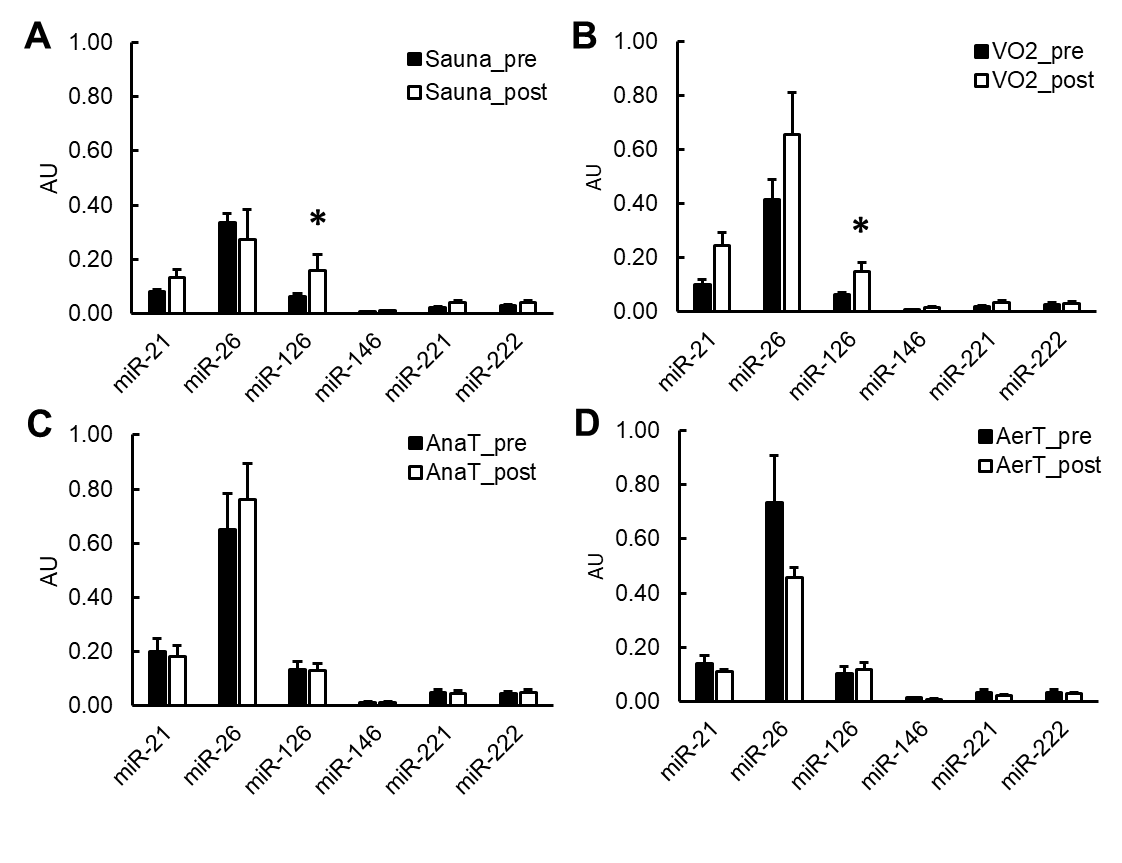
**Supplementary Figure 3.** miR expression in leucocytes before and after control (sauna) or exercise protocols (n=6-8). The expression of miR-126 was increased after control (sauna) and VO2max test protocols (A,B) and miR-21 expression increased after VO2max test (B). There were no changes in the rest of the studied miRs in any of the exercise protocols. *p≤0.050. Figure shows mean+SEM.


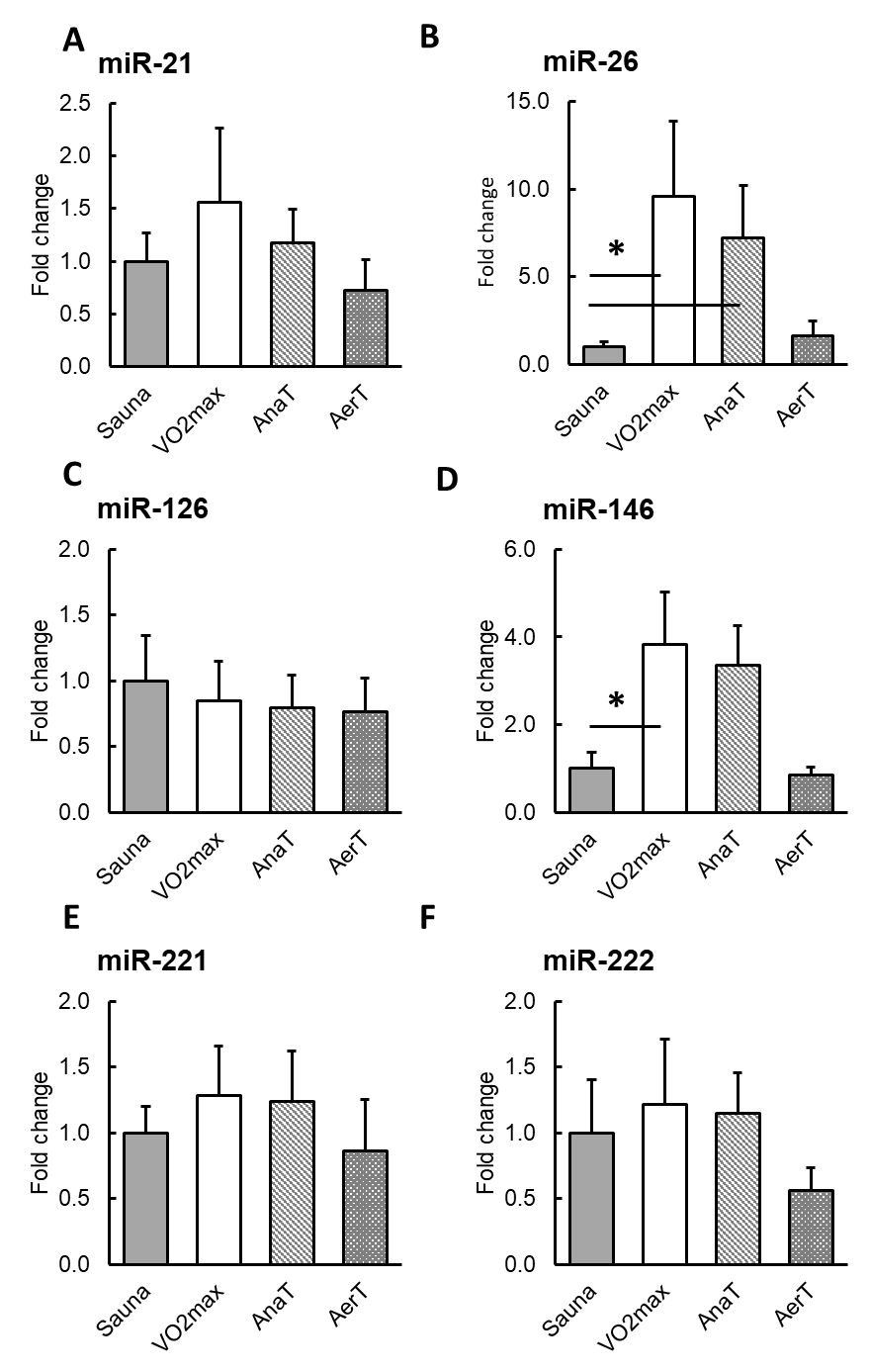


**Supplementary Figure 4.** miR expression in sweat non-EV fraction in control (sauna) or endurance exercise tests (n=4-8). There was an increase in miR-26 (B) and miR-146 (D) levels after VO_2max_ and in miR-26 after AnaT test compared with sauna control. There were no significant changes in the studied miRs compared with sauna in any of the exercise tests. Figure shows mean+SEM.


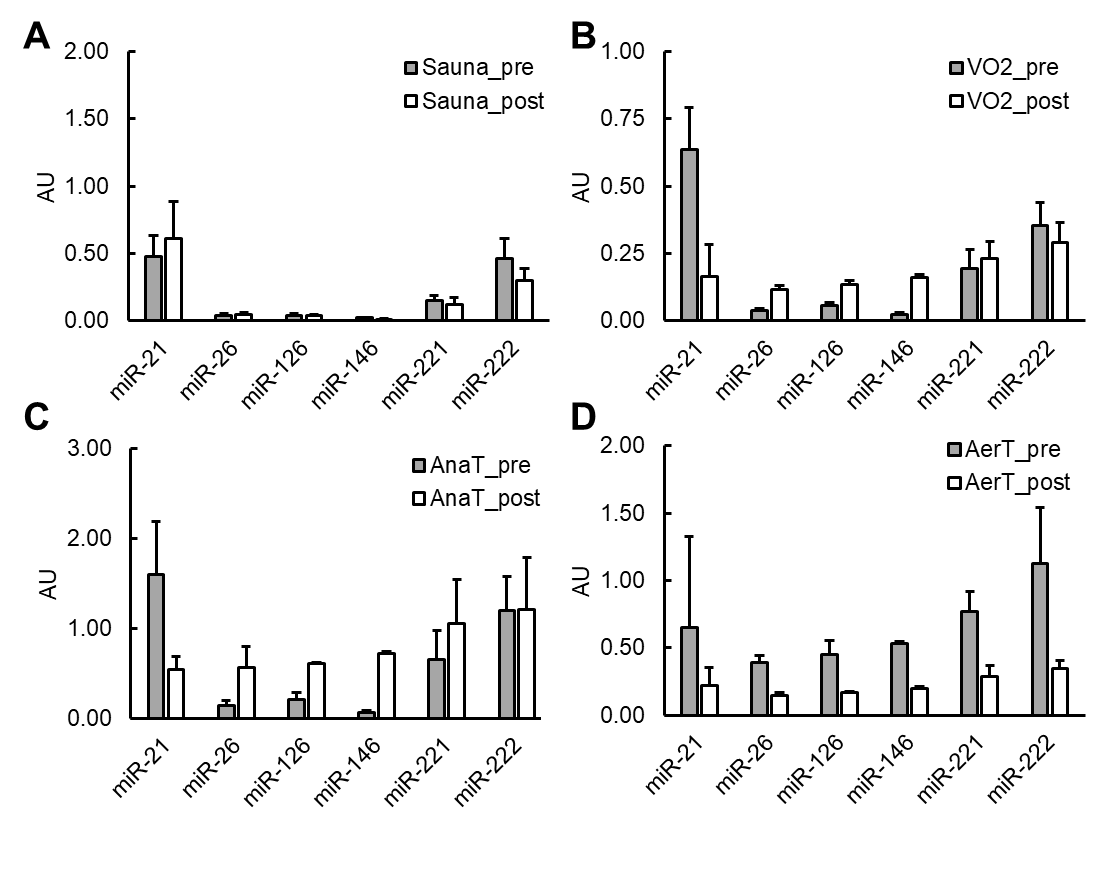
**Supplementary Figure 5.** miR expression in serum non-EV fraction before and after control (sauna) or endurance exercise tests (n=5-8). There were no significant changes in the miR levels before and after control (sauna) or exercise tests. Figure shows mean+SEM.
